# FAIR in action - a flexible framework to guide FAIRification

**DOI:** 10.1038/s41597-023-02167-2

**Published:** 2023-05-19

**Authors:** Danielle Welter, Nick Juty, Philippe Rocca-Serra, Fuqi Xu, David Henderson, Wei Gu, Jolanda Strubel, Robert T. Giessmann, Ibrahim Emam, Yojana Gadiya, Tooba Abbassi-Daloii, Ebtisam Alharbi, Alasdair J. G. Gray, Melanie Courtot, Philip Gribbon, Vassilios Ioannidis, Dorothy S. Reilly, Nick Lynch, Jan-Willem Boiten, Venkata Satagopam, Carole Goble, Susanna-Assunta Sansone, Tony Burdett

**Affiliations:** 1 Luxembourg Centre for Systems Biomedicine, ELIXIR Luxembourg, University of Luxembourg, L-4367 Belval, Luxembourg; 2 University of Manchester, Department of Computer Science, The University of Manchester, Manchester, M13 9PL UK; 3 Oxford e-Research Centre, Department of Engineering Science, University of Oxford, 7 Keble Road, OX13QG Oxford, UK; 4 European Molecular Biology Laboratory, European Bioinformatics Institute (EMBL-EBI), Hinxton, CB10 1SD UK; 5 Bayer AG, Business Development & Licensing & OI, Muellerstrasse 178, 13353 Berlin, Germany; 6 The Hyve BV, Arthur van Schendelstraat 650, 3511 MJ Utrecht, The Netherlands; 7 Institute for Globally Distributed Open Research and Education (IGDORE), Gothenburg, Sweden; 8 Data Science Institute, Imperial College, London, UK; 9 Fraunhofer Institute for Translational Medicine and Pharmacology (ITMP) and Fraunhofer Cluster of Excellence for Immune Mediated Diseases (CIMD), Schnackenburgallee 114, 22525 Hamburg, and Theodor Stern Kai 7, 60590 Frankfurt, Germany; 10 Department of Bioinformatics (BiGCaT), NUTRIM, FHML, Maastricht University, Maastricht, The Netherlands; 11 College of Computer and Information Systems, Umm Al-Qura University, Mecca, Saudi Arabia; 12 Department of Computer Science, Heriot-Watt University, Edinburgh, EH14 4AS Scotland UK; 13 Ontario Institute for Cancer Research MaRS Centre, 661 University Avenue, Suite 510, Toronto, Ontario M5G 0A3 Canada; 14 Vital-IT Group, SIB Swiss Institute of Bioinformatics, 1015 Lausanne, Switzerland; 15 Novartis Institutes for BioMedical Research, Novartis Pharma AG, Basel, Switzerland; 16 OpenPhacts Foundation, Cambridge, UK; 17 Foundation Lygature, Utrecht, Netherlands

**Keywords:** Research data, Data integration, Data processing, Data publication and archiving

## Abstract

The COVID-19 pandemic has highlighted the need for FAIR (Findable, Accessible, Interoperable, and Reusable) data more than any other scientific challenge to date. We developed a flexible, multi-level, domain-agnostic FAIRification framework, providing practical guidance to improve the FAIRness for both existing and future clinical and molecular datasets. We validated the framework in collaboration with several major public-private partnership projects, demonstrating and delivering improvements across all aspects of FAIR and across a variety of datasets and their contexts. We therefore managed to establish the reproducibility and far-reaching applicability of our approach to FAIRification tasks.

## Introduction

The past two years have exposed how critical interoperability of data and systems are to society in times of crisis. The deadly COVID-19 pandemic has made people acutely aware of the weak points that have been known to data management experts for a long time: service incompatibilities, data access restrictions, unavailability of data, missing data, and incomplete, ambiguous or absent metadata. These deficiencies have plagued the scientific endeavour, in both academia and industry, and have hampered the management of the COVID-19 crisis in the early stages, from lack of transparency on data provenance (https://www.bbc.com/news/technology-54423988) to difficulty of data sharing, both due to the sensitive nature of personal health data and interoperability issues between different data sources^1,2^. These issues brought to the forefront the call to arms made in the 2016 publication about the “FAIR (Findable, Accessible, Interoperable, Reusable) Data Principles”, in which Wilkinson and colleagues^3^ highlighted with an elegant acronym how life sciences data and services should be improved in order to build an infrastructure for the 21st century.

So successful was the initiative, that it was incorporated into the G20 Leaders’ Communiqué from the Hangzhou Summit (https://ec.europa.eu/commission/presscorner/detail/en/STATEMENT_16_2967) and made a priority by many research funding organisations, including the Horizon 2020 programme of the European Commission (https://ec.europa.eu/research/participants/data/ref/h2020/grants_manual/hi/oa_pilot/h2020-hi-oa-data-mgt_en.pdf). Despite uptake at the policy level and the known benefits of the FAIR principles, detailed technical guidance towards their implementation is still lacking. Feedback from the data management frontlines indicates that there is a significant demand for hands-on, practical advice on how to translate general and high-level FAIR principles into actionable, “tried and tested” processes.

This manuscript describes a “FAIRification framework” designed to address this demand by supporting organisations and projects undertaking a FAIR transformation. Specifically, we describe a reproducible and sustainable process that can be used to improve the adoption of the FAIR principles by optimising the use of available resources and expanding organisational FAIR data management capabilities. This is achieved through focused prioritisation of needs, based on a thorough analysis of the unique and specific FAIR challenges of each specific project. This framework is one of the outcomes of the FAIRplus consortium (https://fairplus-project.eu), an international project with partners from academia and major pharmaceutical companies, funded by the Innovative Medicines Initiative (IMI, https://www.imi.europa.eu), the largest private-public partnership program funding health research and innovation.

## Results

**Fig. 1:**
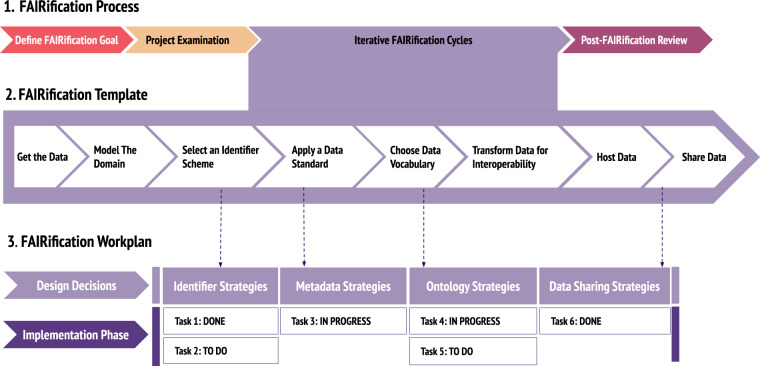
The three components of the FAIRification Framework: the conceptual FAIRification Process, the FAIRification Template covering all aspects of FAIRification and the FAIRification Workplan as a single tailored implementation guide.

Our FAIRification framework (https://w3id.org/faircookbook/FCB079) consists of three distinct components, shown in Fig. 1: a reusable FAIRification Process, which outlines the main phases of a FAIRification activity; a FAIRification Template, which breaks down key elements of the process into a series of steps to follow when undertaking a FAIR transformation; and a FAIRification Workplan layout, which provides a structure for organising FAIR implementation work tailored to the needs of a specific project. Our FAIRification framework was developed in collaboration with over 17 IMI data-producing research projects^4^ (full list in Supplementary Table 1). Throughout these numerous collaborations, we applied this framework to clinical interventional study datasets, data generated in the laboratory to elucidate molecular interactions, as well as real-world and clinical observational data. However, the framework is generalizable to any dataset, as well as other disciplines beyond the life sciences. Its power lies in providing a process that is reproducible over and over, making it a sustainable solution for any organisation looking to improve its FAIR data processes. The framework is also agnostic of any specific implementation solutions or methodologies, allowing users to leverage the resources already at their disposal rather than being forced to invest in solutions that may not be right for them.

Importantly, whilst we describe a reproducible framework for undertaking a FAIR transformation, we do not seek to demonstrate that our framework provides the best possible approach to FAIRification in all contexts: comparison of FAIRification results across projects and domains is a complex activity outside the scope of this work. Furthermore, although there exist a small number of FAIRification processes, such as the GO FAIR Three-point FAIRification Framework (https://www.go-fair.org/how-to-go-fair/), these tend to focus on adoption of a specific technical stack (FAIR Data Points in the case of the GO FAIR framework), rather than providing a comparable guided transformation, and as such results cannot be directly evaluated.

### The four phases of the FAIRification process

**Fig. 2:**
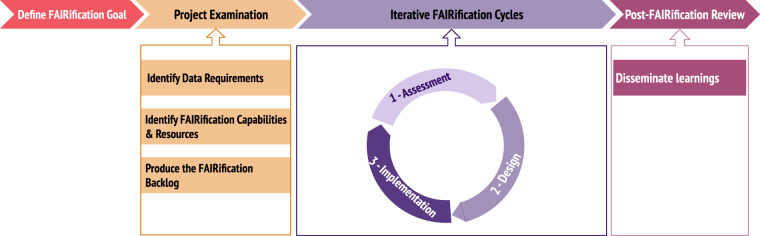
FAIRification Process composed of four distinct phases. This is a reduced version of the process diagram. The full version, with additional explanatory text, is available in Supplementary Fig. 2.

The FAIRification Process, outlined in Fig. 2, describes the general steps that should be followed when engaging in any FAIRification activity. It consists of four distinct phases: a goal definition phase, an initial project examination phase, an iterative cyclical FAIRification phase and a post-FAIRification review.

**Fig. 3:**
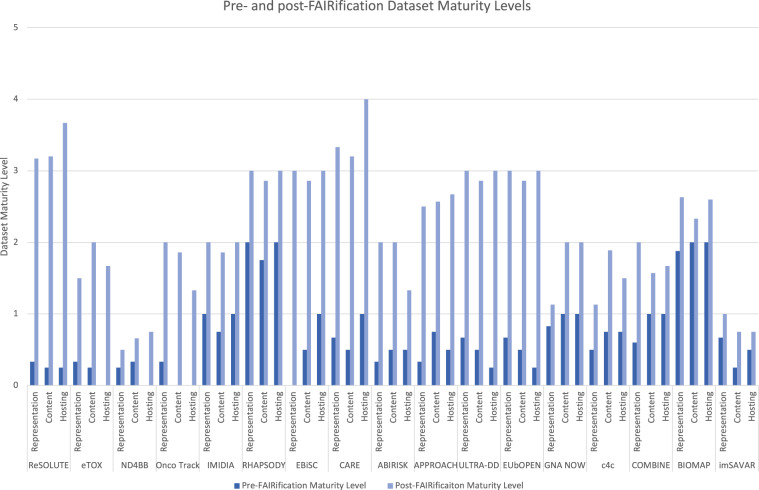
Dataset maturity levels for 17 projects before and after passing through the FAIRification Process. Maturity levels are broken down into representation-related, content-related and hosting-related maturity. The assessments were performed using the FAIR Dataset Maturity (FAIR-DSM) model indicators developed by FAIRplus. It is important to note that maturity improvements should not be compared between projects as they are highly dependent on the specific characteristics of each dataset and the chosen FAIRification goals.

To validate the reproducibility of the process we developed, we evaluated FAIRness improvements for the datasets from the participating IMI projects by comparing dataset maturity^5^ (see Methods and https://fairplus.github.io/Data-Maturity/) before and after FAIRification. A summary of the evaluation, shown in Fig. 3, clearly indicates that FAIRness and maturity improved for all projects that were subjected to our methodology. It is however important to note that maturity levels should not be used to compare across different projects as results depend on a number of factors that can be highly specific to individual projects and datasets, with some indicators not being applicable to all projects. The indicators should serve only to highlight areas for improvement prior to FAIRification and provide an illustration of the scale and impact of the improvements once implemented, for a single project.

### Phase 1: Set realistic and practical goals

Before any FAIRification work is undertaken, it is necessary to determine the desired usability of the data that is not achievable in its current state. From this, one or more clear and precise FAIRification goals can be determined. Based on our experience with the IMI projects, aiming to improve every aspect of FAIR is neither useful nor desirable. All FAIRification efforts come at a cost but may not yield equal levels of benefit^6^. We therefore recommend defining an acceptable “FAIR enough” end state for a dataset whereby key required capabilities are obtained while smaller, less useful enhancements are disregarded. Our experience also suggests that good FAIRification goals should have a defined scope and clearly state how the work will improve scientific value, as well as be specific and actionable.

As visualised in Fig. 3, the CARE (http://www.imi.europa.eu/projects-results/project-factsheets/care) dataset increased in maturity from level 0 or “single-use data level”, to level 3, or “community level”, thanks to a clear objective of improving access to data and its overall discoverability: “*To make the project’s bioactivity data comply with community standards and publicly available so that other researchers can easily reuse the data without repeating the compound identification and testing work*.”. This goal clearly states an aim (compliance with community standards and public availability of data), a scope (the bioactivity data), and the expected scientific value (easily reuse the data without repeating the compound identification and testing work). The CARE aims were implemented in targeted interventions, such as adding an explicit license to the dataset and submitting data to ChEMBL^7^, an international chemical and bioassay repository that generates FAIR-compliant (i.e. globally unique, persistent and resolvable) identifiers and indexed searchable metadata.

We recommend avoiding the word “FAIR” and its derivatives in goals entirely as it is too general to impart clear meaning, e.g. “*FAIRify data resource for public release on project platforms*”. Unlike CARE’s goal, this one does not specify the aim or scope of the work such as compliance with a community standard, mapping to controlled vocabularies or assignment of unique, persistent identifiers. The scientific value is purely implicit - submission to public platforms would likely increase findability - but is not explicitly stated. Finally, FAIRification goals should not factor in implementation details such as how the goal will be accomplished or implemented in terms of resource availability and technologies. These will be considered in the next phase.

### Phase 2: Carefully examine data, capability and resource requirements

Alongside and following the goal-setting step, we identified the need for a project examination process. From early pilot projects such as ND4bb (http://www.imi.europa.eu/projects-results/project-factsheets/nd4bb) and Onco Track (http://www.imi.europa.eu/projects-results/project-factsheets/onco-track), we learned that FAIRification was difficult to accomplish successfully if project capabilities and resources were not fully understood from the outset. For example, goals relating to data hosting improvements cannot be fulfilled if data is not available or accessible, or if the project partners have not reached an agreement on the appropriate licensing and data use conditions. Similarly, goals targeting the annotation of data with open terminologies are only implementable if the data is sufficiently well understood to identify suitable controlled vocabularies and ontologies, and if expertise is available to perform the annotation to a sufficiently high standard.

Tasks related to project examination can be broken down into two distinct categories. Requirements related to the characterization of data such as data types, identifiers, metadata and data standards are categorized as “data requirements” tasks. These tasks are expected to have varying levels of complexity depending on the maturity level targeted for the dataset. Identifying the characteristics that a FAIR dataset should exhibit based on the previously defined FAIRification goal, such as conforming to a specific community standard, has been explicitly added as part of the project examination phase of the FAIRification process.

The tasks in the second category are related to the capabilities that a FAIR data management environment should exhibit to enable and support the realization of a FAIR dataset. These tasks are categorized as “FAIRification capabilities and resources” and include considerations such as data access, data hosting, ontology services and data sharing amongst others. These capabilities are also expected to vary depending on the level of maturity achieved or targeted.

The project examination phase also represents the target phase to employ the FAIR assessment methodology of choice to quantify the level of FAIRness exhibited by the data based on its current characteristics and environment. The assessment outcomes then help shape the necessary steps and requirements needed to achieve the desired FAIRification endpoint.

### Phase 3: Assess, design, implement - then iterate

The practical part of the process centres around the FAIRification cycle, which consists of three separate stages: assessment, design and implementation. This phase typically consists of multiple FAIRification cycles applied in an iterative fashion. Each FAIRification cycle focuses on a single FAIRification goal. We observed that three-month cycles provided the balance that delivered the best results. Three months allows for sufficient time for small, cross-functional teams to deliver observable improvements towards the overall goal, whilst balancing the need for regular validation through assessment. Three-month cycles also ensured a tight focus in work planning, mitigating the risk of insufficiently well-defined implementation tasks that we observed with longer cycles.

An assessment step sits both at the start and the end of each cycle, with the output assessment of one cycle usually serving as input to the next one. For the first cycle, the assessment will usually have been completed as part of the project examination phase. During the design stage, concrete steps from the FAIRification template are identified to achieve the FAIRification goal identified for this cycle. These steps form the FAIRification workplan to be realised during the implementation stage.

### Phase 4: Review against the goals

In this final phase, the cumulative outputs of all the FAIRification processes are reviewed against the initial project goals to assess the overall success of the process. We shaped this stage in a fashion similar to the peer review process employed by academic publications, with individuals not directly involved in the practical implementation work but familiar with the overall data reviewing of the outcomes of the FAIRification work against the initial goals. We identified the need for this because it sets a clear endpoint for the FAIRification work as well as providing independent feedback on the effectiveness of the tasks. Without the review phase, there is a danger of work continuing beyond the point where the benefits to the project justify the continued expenditure of resources.

### The FAIRification template

**Fig. 4:**
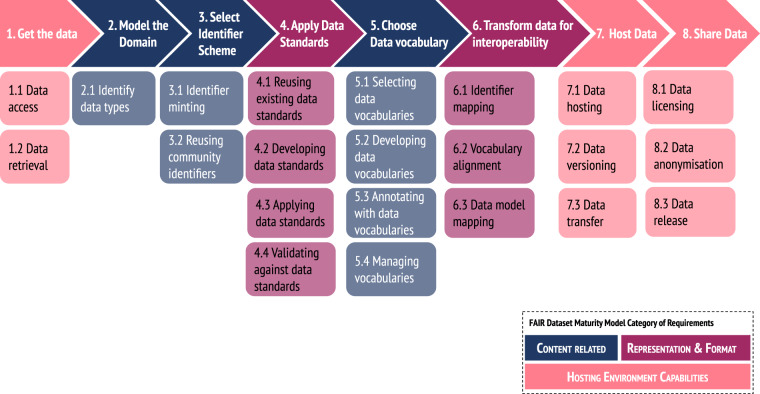
The FAIRification template steps. Each step is colour-coded based on whether its implementation applies to data hosting, representation and format or data content. Each step is broken down into one or more sub-steps. More details can be found in Supplementary Table 2.

The FAIRification Template, shown in Fig. 4, implements the FAIRification Process by providing a set of clear and distinct steps for the implementation stage in the FAIRification cycle phase of the process. The template consists of eight steps relating to data hosting, such as data retrieval and data versioning, to data representation and format, such as applying data standards and vocabulary alignment, and to data content, such as identifier minting and annotation with vocabularies. A more detailed explanation of each step in the template can be found in Supplementary Table 2.

### The FAIRification workplan

The FAIRification Workplan is a specific design and implementation plan customised to an individual project by selecting the template elements required to achieve the intended FAIRification goals according to the project examination. An example of a FAIRification workplan is shown in Supplementary Fig. 1.

For many of the steps in the workplan, solutions may already exist, in the shape of FAIR Cookbook^8,9^ recipes (https://faircookbook.elixir-europe.org), which serve as a guide. Supplementary Table 2 provides links to the recipes that address specific implementation considerations. Once the workplan has been designed, it is put into action within the agreed cycle time frame by either following the steps from an existing recipe or implementing a novel solution, which should then ideally be documented as a new Cookbook recipe, helping build content for others in future who face similar issues.

## Discussion

Whilst developing our FAIRification process, we learned three key lessons and summarised these into take-home messages, in Box 1.

Developing the FAIRification framework was an iterative process: a journey we tailored to the IMI projects’ real data needs and scenarios^4^. To clarify the fundamental steps of the FAIRification process, we built on the prior state of the art described by Jacobsen and colleagues^10^, work undertaken by the EHDEN project (http://www.imi.europa.eu/projects-results/project-factsheets/ehden), an IMI sibling project in the area of health data research, and by the Pistoia Alliance with the FAIRtoolkit (https://fairtoolkit.pistoiaalliance.org/), refining and expanding as needed to shape it to fit specific requirements, while remaining compatible with community practice, for instance as outlined by the GO-FAIR initiative^11^. Guidance on suitable criteria for evaluating the costs and benefits of performing FAIRification tasks on any dataset or project, in particular for the retrospective FAIRification of existing data, is discussed elsewhere^6^ and lies outside the scope of the FAIRification framework.

The FAIRification process was initially presented as a linear workflow focusing on the tasks that are involved in the FAIRification of a dataset. It progressively evolved into the current process with a core iterative component to reflect the cyclic nature of improving a dataset’s FAIRness and maturity levels as well as evolving FAIRification capabilities. Another example is the composition of the FAIRification framework, which initially consisted of a single level with the elements that are now part of the template. The abstraction of the process took a step back from the implementation considerations inherent in the template, while the development of a workplan from the template emerged as a natural consequence of the design and implementation phases of the FAIRification cycle, which involved picking the specific steps and sub-steps required to achieve the FAIRification goal. The distinction between template and workplan also helped to communicate to data owners that there is a clear path from FAIRification goals to the tasks and steps required to reach a higher level of maturity.

The successful development and implementation of a given FAIRification workplan are only possible through the assembly of a multidisciplinary team. Required roles and skills depend of course on the nature of the project and FAIRification goals but can include data managers or stewards, ontologists, curators, data scientists, software developers, system administrators and project managers. In particular, the implementation of FAIR solutions often requires technical skills such as ontology engineering, building “extract, transform and load” (ETL) procedures or designing FAIR-compliant data hosting solutions. Identifying the most suitable areas for improvements and thus the definition of FAIRification goals requires an in-depth understanding of the structure and content of the data, its representation and hosting requirements. This can only be achieved through close interaction with the data and a complete understanding of the lifecycle of the dataset.

A number of our FAIRification efforts were hampered and delayed by difficulties to set up appropriate legal agreements to arrange data access due to restrictive and complex data sharing policies and by insufficient buy-in from data owners due to lack of personnel, knowledge and budget available for legacy projects. Data licensing and data availability are key elements of the FAIR principles and should therefore always receive due consideration. This experience echoes that of IMI eTRIKS (http://www.imi.europa.eu/projects-results/project-factsheets/etriks), which reported similar issues^12^.

The early stages of developing the FAIRification process made use of an extensively documented previous study^13^ and four very different pilot projects to test the initial steps and assumptions of the process. One pilot (http://w3id.org/faircookbook/FCB045) project dataset used, the ReSOLUTE (Research Empowerment on Solute Carriers, http://www.imi.europa.eu/projects-results/project-factsheets/resolute) transcriptomics dataset, was publicly available in the SRA archive with additional information about the cell cultures and cell lines provided in separate PDF files, which is hard to efficiently extract and reuse. To improve the data findability, curators developed ETL procedures that mined experimental details from PDF files and enriched metadata about cell cultures. These sample descriptions, annotated with ontology terms enabling ontology-powered searching, were validated against the MINSEQE minimum metadata checklist^14^ (https://fairsharing.org/FAIRsharing.a55z32) in order to ensure that they met broader community standards, and submitted to the Biosamples database^15^. The cell line sample metadata was also cross-referenced to the corresponding Cellosaurus^16^ ID to link them to the Cellosaurus knowledge base for easier data interpretation.

This yielded a number of learning points. Firstly, retrospectively achieving compliance with “community reporting guidelines” (see for example https://fairsharing.org/search?recordType=reporting_guideline) can be challenging owing to the need to interpret a narrative rather than being able to access machine-actionable data^17^. Second, some leading archives rely on earlier-generation models which provide little support for ontologies and semantic annotations, which hampers interoperability and findability. Finally, engagement from the data owners is essential to maximise FAIRification gains.

In addition to the direct project interactions, some of the pharma partners in FAIRplus also trialled the framework through a range of specific FAIRification objectives, which provides some evidence for the broad applicability of our FAIRification process. One use case revolved around enhancing interoperability by developing an application ontology to integrate multi-omics data from independent sources, a challenge faced by the Boehringer-Ingelheim partner. Their proposed solution made extensive use of a specific FAIR Cookbook recipe (https://w3id.org/faircookbook/FCB023). Another challenge, from the AstraZeneca partner, focused on a FAIRification goal looking at expressing “allowed data use” in a way compatible with a DCAT-based data catalogue to increase findability and reusability. The output of the work yielded a new content type in the FAIR Cookbook (https://w3id.org/faircookbook/FCB035).

Although much progress has been made to make the FAIR principles tangible by providing concrete examples, there is neither a single one-size-fits-all approach to realising FAIR in the life sciences in general nor a community-wide consensus on a FAIR representation of any given data type. The FAIRification framework provides a valuable tool to guide FAIRification efforts in a range of communities and for a variety of data types. There already exists a wide range of tools, standards, indicators and measures developed to improve data FAIRification practice, such as FAIRness assessment frameworks proposed by the RDA^18^ or FAIRsFAIR^19^, the Data Use Ontology (DUO)^20^ standard for encoding data reuse conditions or the biosciences specific resource markup framework, Bioschemas^21^. The framework is agnostic of any specific indicators or implementation and any of these can be plugged into the framework in the relevant places.

The successful application of the framework in both exemplar projects and its integration into the working processes of several pharma partners demonstrates its broad applicability to any life sciences data. Supported by an active and knowledgeable community passionate about the importance of bringing FAIR to the forefront of scientific data management, it should serve as a guide to anyone looking to address FAIRification challenges.

Box 1 Take-home messages 1. **Focus on incrementally achievable targets**. Projects approach the FAIR principles in different orders and risk overdoing. Instead, we focus on achieving the elements of FAIRness that matter most to the needs of the project to reach a balanced “FAIR enough” status.

2. **Tailor the FAIRification process to individual needs**. Projects have different needs, even when the underlying data, capability and resource requirements appear to be quite similar. Customising the relevant template elements allows the formation of a coherent workplan.

3. **Assemble a multi-disciplinary team**. Projects are often multi-partner, international and distributed in nature, with datasets of different provenance and source. A successful FAIRification process starts with bringing together diverse teams that include the data owners as well as professionals who can tackle the legal, curational and technical infrastructure aspects.

## Methods

### Incremental framework development

The development of the FAIRification framework was an iterative process. It was developed over the course of two years, starting with a set of four pilot projects whose FAIRification served to establish the basic structure and elements of the process. This was then refined over several iterations, using a wide range of IMI projects as well as FAIRification use cases elicited from EFPIA partners, to establish the framework described above.

Both the pilot projects and subsequent projects were selected from the full pool of IMI projects through a formal process. The details of this process and how it was established are discussed elsewhere^6^. Once selected, projects were passed on to cross-disciplinary working groups who worked with the data owners to set FAIRification goals and progress the project through the steps and stages of the framework.

The FAIRification template was developed to accommodate a wide range of projects and data types. The steps and sub-steps of the FAIRification template were refined from data FAIRification efforts and experience in a wide range of contexts and domains and from the prior experience of cross-disciplinary task teams within the FAIRplus project (see below). Elements of the template are more relevant to some areas than others but overall, the template can be applied and customised to any type of project, rather than being applicable to only very specific data types, such as healthcare or clinical trial data.

### Cross-disciplinary task teams

The practical work executed during the FAIRplus project was carried out by cross-disciplinary teams, referred to as ‘Squads’ (to borrow the terminology of the Spotify model described in https://www.atlassian.com/agile/agile-at-scale/spotify), assembled to provide the expertise required for a given task. The working practices and methodology of these squad teams were iteratively refined over 2 years, and a report on this process is in preparation. The personnel were recruited to squads from across all work packages, allowing the incorporation of specialist knowledge, and fostering information exchange within the project. Besides this base composition, other floating team members were recruited to address specific and arising needs in the short term, allowing a flexible and tailored response. Squad representatives engaged early in project discussions between IMI project representatives and FAIRplus triage staff, allowing a preview of the types of data and issues that may be coming through the onboarding pipeline, and determining whether potential areas of work could improve the content of the FAIR Cookbook. This also allowed a level of expectation management regarding the distribution of work for the actual FAIRification tasks between project representatives and FAIRplus personnel, as well as the development of a collaborative relationship with external project representatives. During these exchanges, the squads would engage with project representatives to identify tasks and goals that were realistically achievable in the given time frames, routinely being of roughly 3 months total duration.

### Validation process

In parallel to the development of the FAIRifiation framework, we also developed a FAIR DataSet Maturity (FAIR-DSM) Model (https://fairplus.github.io/Data-Maturity/). This allowed us to assess the maturity of the datasets used to validate the FAIRification process prior to and following any FAIRification work. In the initial stages of the framework development, we made use of existing approaches including the RDA indicators^18^ and the FAIRsFAIR indicators^19^ to evaluate FAIRifiation improvements. While these alternative models demonstrated satisfactory results, they generally treat each element or principle of FAIR as a stand-alone element. The FAIR-DSM on the other hand evaluates a dataset as a whole, providing a more balanced view of its overall maturity in terms of content, representation and hosting.

**Fig. 5:**
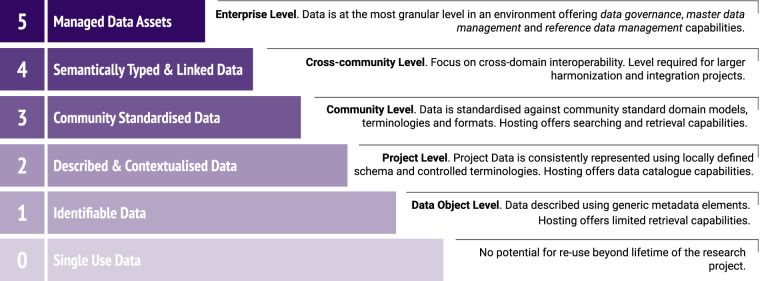
FAIR-DSM levels. The 5 levels of the FAIR-DSM as well as the additional “level 0” baseline for single-use data, with a brief description of the data characteristics and capabilities required for each level.

The FAIR-DSM is described in detail elsewhere^5^ but briefly, it consists of 5 maturity levels characterised by increasing requirements across all aspects of FAIR, plus a reference level, referred to as “level 0”, representing a state of data that is missing most or all fundamental FAIR requirements. The model considers 3 categories of requirements: content-related requirements; representation and format requirements; and hosting environment capabilities. In order to conform to a given level of the model, a dataset needs to fulfil a set of indicators covering the requirements for each of the 3 categories at this level. Figure 5 provides a summary description and perspective for each level.

### Ancillary materials

#### The FAIR cookbook

The learnings and insights gained from the efforts of the FAIRplus project were distilled into individual “recipes” making up the FAIR Cookbook. This specific practical guidance is intended to provide concrete solutions to common FAIR data problems. The FAIR Cookbook is available at https://faircookbook.elixir-europe.org/.

#### The FAIR Wizard, mining the FAIR Cookbook

We recognize that providing guidance that leads to a specific implementation of the FAIRification process is still an expert activity. To provide support for those who are newer to FAIR implementation, we have begun developing a “FAIR Wizard” (https://www.ebi.ac.uk/ait/fair-wizard/) to facilitate the work of project managers and data scientists in identifying FAIRification goals, examining projects and developing FAIRification solutions. The wizard as a whole is effectively based on the FAIRification template, with the output provided to a user representing a skeleton FAIRification workplan.

The FAIR wizard collects FAIRification goals from datasets that we worked with and the knowledge consolidated in the FAIR Cookbook in the form of curated solutions for the common use cases, which can be reused directly. It also assists people in identifying their own use cases through a series of questions and FAIR assessments and proposes solutions accordingly.

The FAIR wizard utilises FAIRification resources developed by this project and other platforms, suggests FAIRification materials based on the FAIRification requirements, and designs FAIRification solutions for data owners, data stewards and other people involved in FAIRification.

#### The IMI data catalog

All datasets engaged during the establishment and validation of the FAIRification framework were included in the IMI Data Catalog^22^ (https://datacatalog.elixir-luxembourg.org/) hosted by ELIXIR Luxembourg. Dataset entries include information on the maturity level of the dataset before and after FAIRification efforts as well as key metadata about the project, experimental process and dataset.

## Supplementary information

Supplementary materials

## Data Availability

• IMI Data Catalog: https://datacatalog.elixir-luxembourg.org/ • EBI repositories ◦ ReSOLUTE data in BioSamples: https://www.ebi.ac.uk/biosamples/samples?filter=attr:project:RESOLUTE ◦ CARE ■ Pre-FAIRification: https://zenodo.org/record/5872683#.YvZff-xBwbk ■ FAIRified data in ChEMBL: 10.6019/CHEMBL4651402 • FAIRification results: https://fairplus.github.io/fairification-results/

## Code availability

• FAIRplus Github organisation: https://github.com/FAIRplus

• FAIR Maturity Model: https://github.com/FAIRplus/Data-Maturity (MIT license)

• FAIR Wizard: https://github.com/FAIRplus/FAIR_wizard (Apache-2.0)

• FAIR Cookbook: https://github.com/FAIRplus/the-fair-cookbook (CC BY 4.0)

• IMI Data Catalog: https://github.com/FAIRplus/imi-data-catalogue (AGPL-3.0 for code, CC BY-NC-SA 4.0 for data)
