# Supplementary material for: FAIR in action - a flexible framework to guide FAIRification
Source: Sci Data. 2023 May 19;10:291. doi: 10.1038/s41597-023-02167-2 (PMC10199076; doi:10.1038/s41597-023-02167-2)
Supplement: Supplementary file 1 — Supplementary materials [file 41597_2023_2167_MOESM1_ESM.pdf]

“FAIR in action” supplementary materials

Table of contents

- Supplementary Table 1 - IMI projects that interacted with FAIRplus
- Supplementary Table 2 - steps of the FAIRification template
- Supplementary Figure 1 - FAIRification Workplan example for the CARE project
- Supplementary Figure 2 - FAIRification Process diagram, full version

Supplementary Table 1 - IMI projects that interacted with FAIRplus

Project	Engagement phase	Data types	FAIRification goal(s)	Outputs/recipes
Onco Track	Pilot project	Patient-derived samples (oncology)	Convert the Onco Track sample metadata to a structured and consistent data format, improves the findability, interoperability, and reusability of the metadata	FCB044
ND4BB	Pilot project	in-vitro data on compound properties for known antibiotics	Creating a FAIR & machine-readable data set of the AMR database	FCB043
eTOX	Pilot project	Chemical compounds, toxicology assays	Semantic markup to reduce free text descriptors	FCB042
ReSOLUTE	Pilot project	Transcriptomics, proteomics, metabolomics	1. Deposition to a public repository and compliance to community standard (MINSEQE)<br>2. Conversion from proprietary to open format	FCB045; FCB029
IMIDIA	FAIRification process v2.0	Clinical data, transcriptomics	1. Identify gaps in current metadata annotations and pick the best ontologies to fill them<br>2. Make metadata findable/searchable	n/a
RHAPSODY	FAIRification process v2.0	Clinical data, transcriptomics	1. Identify gaps in current metadata annotations and pick the best ontologies to fill them<br>2. Make metadata findable/searchable	n/a
EBiSC I & II	FAIRification process v2.1	Cell line metadata, genomics	EBiSC seeks to make specialised cell lines as findable as possible for its users, based on a selected (small) set of relevant descriptors.	n/a
APPROACH	FAIRification	Clinical trial data,	1. Map the metadata parameters (data dictionary) to	FCB078;

	process v2.1	imaging, biomarkers	appropriate domain-relevant ontologies and standards to enable applying to data catalogues and repositories to make the data more findable.<br>2. Provide advice and information to the consortium members so they can decide on the type of licensing for publicly sharing the data and clarifying the possible reuse of the data.	FCB025
ABIRISK	FAIRification process v2.1	Clinical trial data	1. Map the data dictionary to CDISC and appropriate domain-relevant ontologies to facilitate data interoperability and enable sharing of metadata in data catalogues and repositories to make the data more findable.<br>2. Provide advice and information to the consortium members so they can decide on the type of licensing for publicly sharing the data and clarifying the possible reuse of the data.	FCB078; FCB025
CARE	FAIRification process v2.1	Compound and bioassay data	To publish data in open archives and comply with community data standards so that other researchers can find and reuse the compound and bioassay data.	FCB057
ULTRA-DD	FAIRification process v2.1	High-content screen data	Promote public access, data dissemination and sharing of project datasets	n/a
EUbOPEN	FAIRification process v2.1	High-content screen data, bio-imaging data	EUbOPEN seeks to make multimodal chemical biology assays as findable as possible to facilitate dataset discovery based on a small number of search criteria	FCB067
COMBINE	FAIRification process current version	Bioassay protocol data	Composition of an application ontology to aid in reproducibility of in-vivo bioassay experiments	FCB023; https://github.com/Fraunhofer-ITMP/bpo
c4c	FAIRification process	Clinical trial metadata, eCRFs	Study- and protocol-level additional (meta)data (such as in/exclusion criteria) required alongside the CRF data	In progress

	current version - work ongoing		dictionary to make the overall trial data more findable in relation to this information:<br>● Define & refine list of variables to be collected<br>● Represent protocol-level additional (meta)data in a complementary data model<br>● Define extraction processes for populating variables of interest	
BIOMAP	FAIRification process current version - work ongoing	Clinical trial data, omics data	1. FINDABILITY Improve findability of project metadata for external researcher through publication of metadata in the IMI Data Catalog<br>2. INTEROPERABILITY (Re)Align the data glossary with the OMOP community standard in order to improve the data's interoperability with other OMOP datasets<br>3. REUSABILITY Define and implement QC policies/best practice to ensure that data files can be reached from patient metadata and that data files are in the correct format as defined by the metadata	In progress
GNA NOW	FAIRification process current version - work ongoing	in-vivo and in-vitro efficacy data	Standardization and development of workflows involving data archiving process for terminated sub-projects	In progress
imSAVAR	FAIRification process current version - work ongoing	Omics data	1. Create a data dictionary that is harmonised across species as well as being consistent with the prospective metadata collection form of imSAVAR in order to facilitate data reuse across the imSAVAR project. Includes harmonising/mapping terms against a CV where possible<br>2. Design a metadata template to capture study/protocol-level contextual metadata about the dataset, including antibodies, preparation methods and analysis workflows, in order to improve reusability and data integration	In progress

			3. Suggest data usage conditions to data owner and provide machine-readable sample encoding of conditions for Data Catalog metadata in order to illustrate machine actionable metadata	
ESCulab	FAIRification process current version - work ongoing	Compound and bioassay data	1. Improve the searchability of the data for current and future users for analysis and reuse by enhancing and structuring existing metadata.<br>2. Accessibility: Providing metadata and exposing the data<br>3. Interoperability and Reusability: Improving future interoperability for the project after the timeline of the project<br>4. Reusability: Enable privacy preserving analysis of the data with third parties	In progress
U-BIOPRED	FAIRification process current version - work ongoing	Omics data	*To be determined*	*To be determined*
eTRANSAFE	FAIRification process current version - work ongoing	Chemical toxicity prediction tool	*To be determined*	*To be determined*

Supplementary Table 2 - steps of the FAIRification template

Capabilities domain	General FAIRification step	FAIRification sub-step	Description	Related FAIR Cookbook recipes
Hosting environment capabilities	1. Get the data	1.1 Data access	Considerations relating to how data is accessed, eg through APIs, via controlled access	FCB014, FCB015, FCB073
		1.2 Data retrieval	Considerations relating to data retrieval, eg query language, results representation and exporting capabilities	FCB040, FCB046, FCB060, FCB070
Content-related capabilities	2. Model the domain	2.1 Identify data types	Data type identification informs the selection of appropriate data standards, ontologies and target repositories	FCB027, FCB057
	3. Select the identifier scheme	3.1 Identifier minting	How to create unique, persistent and resolvable identifiers	FCB006, FCB007, FCB008, FCB077
		3.2 Reusing community identifiers	How to reuse existing identifiers in a dataset	FCB016, FCB017
Representation & format capabilities	4. Apply data standards	4.1 Reusing existing data standards	How to reuse existing data standards	FCB025
		4.2 Developing data standards	How to develop a new data standard if no appropriate standards exist	FCB025, FCB026, FCB027
		4.3 Applying data standards	How to apply data standards to datasets, especially retroactively	FCB025, FCB029, FCB078

		4.4 Validating against data standards	How to use validation to ensure that a dataset is compliant with a data standard	FCB028, FCB030
Content-related capabilities	5. Choose data vocabularies	5.1 Selecting data vocabularies	How to select the most appropriate vocabularies to annotate a dataset	FCB019, FCB020
		5.2 Developing data vocabularies	How to develop new vocabularies from scratch	FCB021
		5.3 Annotating with data vocabularies	How to annotate data and metadata with terms from vocabularies	FCB022, FCB023
		5.4 Managing vocabularies	How to manage vocabularies and ontologies	FCB003, FCB004, FCB005, FCB022
Representation & format capabilities	6. Transform data for interoperability	6.1 Identifier mapping	How to map between different types of equivalent identifiers	FCB016, FCB017, FCB018
		6.2 Vocabulary alignment	How to map between different equivalent vocabulary terms	FCB022
		6.3 Data model mapping	How to map equivalent concepts from different data models	FCB016, FCB031, FCB058, FCB059, FCB065
Hosting environment capabilities	7. Host your data	7.1 Data hosting	Considerations around data hosting infrastructure such as markup and search engine optimisation	FCB009, FCB010, FCB011, FCB012, FCB013, FCB047, FCB048
		7.2 Data versioning	Considerations around data versioning	FCB009, FCB036
		7.3 Data transfer	Considerations around data transfer such as file formats, repository types and checksumming	FCB014, FCB015, FCB052, FCB053
	8. Share your	8.1 Data licensing	Data licensing considerations such as	FCB032, FCB033,

	data		which license is most appropriate for a given scenario	FCB034, FCB035, FCB036
		8.2 Data anonymisation	Data anonymisation considerations	n/a - due to the complexity of this subject, incl legal ramifications, the FAIR Cookbook does not include guidance on data anonymisation
		8.3 Data release	Data release considerations such as when to release a dataset and where to release it	FCB009, FCB061, FCB067

Supplementary Figure 1 - FAIRification Workplan example for the CARE project

This figure shows an example of a real FAIRification workplan, as used for the CARE project. The top half of the workplan covers the FAIRification goal, the key findings of the project examination and the results of the pre-FAIRification maturity assessment. The middle section lists the “Design decisions” or high-level tasks required to achieve the stated FAIRification goal, grouped into logical categories. The bottom section shows the progress of the implementation phase, with concrete steps required to complete each of the tasks from the previous section. In this example, all steps are marked in green, indicating that they have been completed. Additional colour coding is available to mark tasks as To Do (orange), In Progress (blue) or At Risk (red). The latter category is used for tasks that might not be completed during the current FAIRification cycle due to circumstances that emerged after the task had been prioritised.

FAIRplus Tailored FAIRification Process - CARE - Iteration 2

1- Define FAIRification Goal

Define desired and expected outcomes of FAIRification.

To publish data in open archives and comply with community data standards so that we share identification and testing of compounds for therapeutic properties.

- **Findability**: Improve Findability of (meta)data by mapping to common data models and ontologies
- **Reusability**: Improve Reusability by encoding data reuse conditions in metadata

2- PROJECT EXAMINATION

Identify Data Requirements

Current	Projected
Consent/Licence form varies based on cohort	Unified license form across CARE
Inconsistent use of identifiers (mixture of internal and other)	Mapping to Standardisied identifiers (InchI and/or SMILEs)
Lab/biomarker results	Standardisation of IC50 values
- Variety of data types<br>- Data in simple tabular format (csv)	Data can be downloaded in various standardised formats (XML, JSON)
Data dictionary not available	Metadata for project added from BAO and CHEMBL-preferred ontologies

Identify Data FAIRification Capabilities

Current	Projected
Data owners need to clarify data access and reuse criteria	Hosting platform selected will expose licenses for sharing rules in place

Identify Data FAIRification Resources

Current	Projected
Data currently only hosted internally on a temporary server or on Zenodo	Data shared in public resource (CHEMBL)
	Project and dataset metadata in IMI Data Catalogue

3- Assessment

Pre-FAIRification Assessment Results

DSM Maturity levels (FAIR DSM indicators v1.0) :

- Maturity level - Representation: 0.67
- Maturity level - Content: 0.5
- Maturity level - Hosting: 1.0

Overall maturity level: 0

NOTE: Ultimately, a single dataset (ChEMBL) was selected for use as an exemplar implementation.

4- Design Decisions

Design identifier strategies

- Map current identifiers (mixture of internal and standard) to InChI and SMILE ids.

Design metadata strategies

- Publish agreed metadata in the IMI Data Catalog (FAIRplus requirement)

Design ontology strategies

- Apply ontologies/standardised terminologies to metadata

Design data sharing strategies

- Support data sharing strategy by encoding data reuse criteria using DUO/ODRL
- Determine suitable data reuse license
- Identify most appropriate hosting platform (various public dbs, single internal repo with DAC, semi-public custom repo)

5- Implementation Phase

TASKS

Tasks LEGEND:

TO DO	*IN PROGRESS*
AT RISK	*DONE*

Identifier strategies:

- *InChi and SMILE identifiers generated (recipe)*
- *ChEMBL IDs assigned in each compound and assay. (Collection ID)*

Metadata strategies:

- *Data mapped to the ChEMBL submission model*
- *Assay value split into numeric values and operators.*
- *Tabular form for collection of assay from wide-short to tidy-long*
- *CARE project metadata submitted to the IMI Data Catalog*

Ontology strategies:

- *ChEMBL model for general bioactivity data (recipe)*
- *BioAssay Ontology(BAO), CHEMINF vocabulaires used for compound annotation*
- *Introduced Cellosaurus ID for cell line (Vero-E6)*

Data sharing strategies:

- *ChEMBL license used for data sharing, CC-SA 3.0*

6- Assessment

Post-FAIRification Assessment Results

DSM Maturity levels (FAIR DSM indicators v1.0) :

- Maturity level - Representation: 3.43
- Maturity level - Content: 3.2
- Maturity level - Hosting: 4.0

Overall maturity level: 3

Supplementary Figure 2 - FAIRification Process diagram, full version

The FAIRification Process, composed of four distinct phases. This is an expanded version of the diagram shown in Figure 2 in the main text.

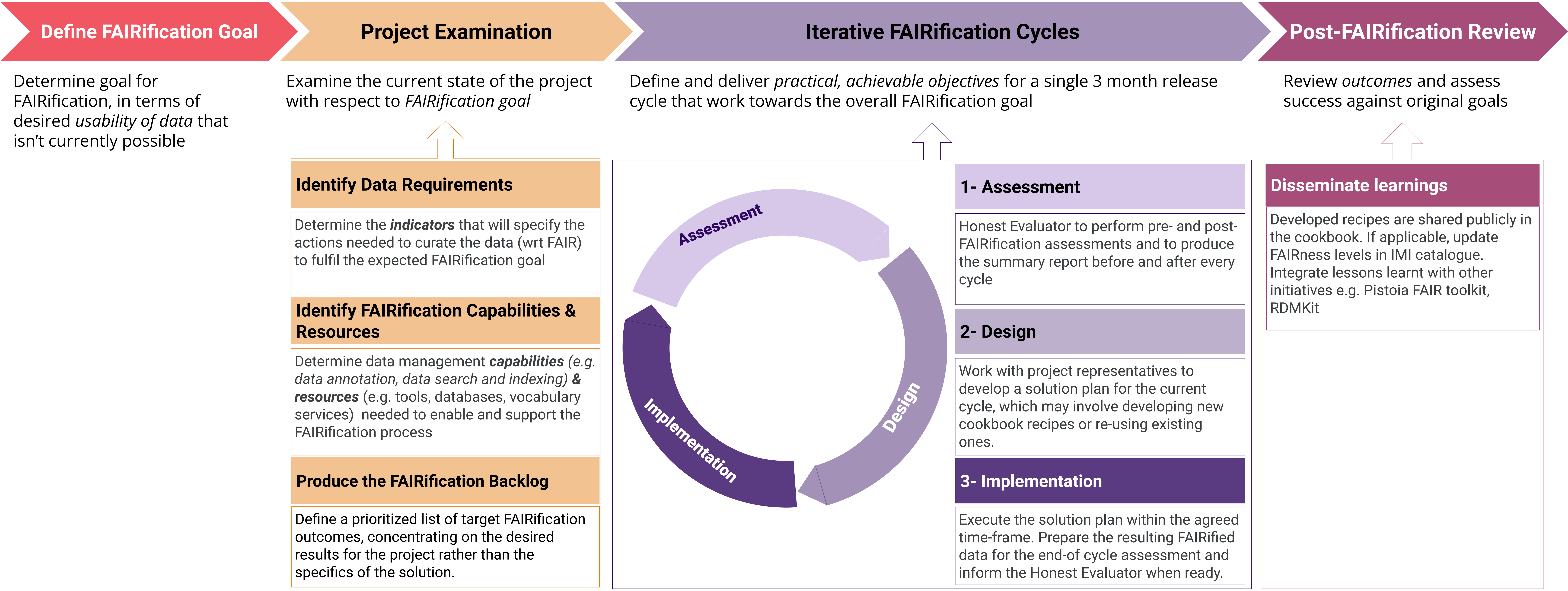
Define FAIRification Goal
Determine goal for FAIRification, in terms of desired *usability of data* that isn't currently possible
Project Examination
Examine the current state of the project with respect to *FAIRification goal*
Identify Data Requirements
Determine the ***indicators*** that will specify the actions needed to curate the data (wrt FAIR) to fulfil the expected FAIRification goal
Identify FAIRification Capabilities & Resources
Determine data management ***capabilities*** *(e.g. data annotation, data search and indexing)* **&** ***resources*** (e.g. tools, databases, vocabulary services) needed to enable and support the FAIRification process
Produce the FAIRification Backlog
Define a prioritized list of target FAIRification outcomes, concentrating on the desired results for the project rather than the specifics of the solution.
Iterative FAIRification Cycles
Define and deliver *practical, achievable objectives* for a single 3 month release cycle that work towards the overall FAIRification goal
Assessment
Design
Implementation
1- Assessment
Honest Evaluator to perform pre- and post-FAIRification assessments and to produce the summary report before and after every cycle
2- Design
Work with project representatives to develop a solution plan for the current cycle, which may involve developing new cookbook recipes or re-using existing ones.
3- Implementation
Execute the solution plan within the agreed time-frame. Prepare the resulting FAIRified data for the end-of cycle assessment and inform the Honest Evaluator when ready.
Post-FAIRification Review
Review *outcomes* and assess success against original goals
Disseminate learnings
Developed recipes are shared publicly in the cookbook. If applicable, update FAIRness levels in IMI catalogue. Integrate lessons learnt with other initiatives e.g. Pistoia FAIR toolkit, RDMKit
